# Wheat yield and nitrogen use efficiency enhancement through poly(aspartic acid)-coated urea in clay loam soil based on a 5-year field trial

**DOI:** 10.3389/fpls.2022.953728

**Published:** 2022-08-30

**Authors:** Peng Yan, Xuerui Dong, Lin Lu, Mengying Fang, Zhengbo Ma, Jialin Du, Zhiqiang Dong

**Affiliations:** ^1^Institute of Crop Sciences, Chinese Academy of Agricultural Sciences, Beijing, China; ^2^Tianjin Agricultural Development Service Center, Tianjin, China

**Keywords:** poly(aspartic acid), winter wheat, yield, nitrogen use efficiency, North China Plain

## Abstract

The innovation of N fertilizer and N management practices is essential to maximize crop yield with fewer N inputs. A long-term field fertilization experiment was established in 2015 on the North China Plain (NCP) to determine the effects of a control treatment (CN) and the eco-friendly material poly(aspartic acid)-coated urea (PN), applied as a one-time basal application method, on winter wheat yield and N use efficiency at four N application rates: 0 (N0), 63 (N63), 125 (N125), and 188 (N188) kg N ha^–1^. The results indicated that compared to CN, PN resulted in a significant increase in wheat yield by 9.6% and 9.2% at N63 and N125, respectively, across the three experimental years, whereas no significant (*p* < 0.05) difference was detected at N188. Leaf area duration (LAD), crop growth rate (CGR), and dry matter accumulation (DMA) increased with increasing N rates, while PN significantly increased LAD and CGR by 5.1%–16.4% and 5.4%–64.3%, respectively, during the anthesis-ripening growth stage and DMA by 13.7% and 10.1% at N63 and N125, respectively, after the anthesis stage compared to CN. During the grain-filling stage, PN significantly increased the kernel maximum grain-filling rate (Gmax) by 21.7% and the kernel weight at the maximum grain-filling rate (Wmax) by 6.7% at N125 compared to CN. Additionally, compared to CN, PN significantly improved the stover and grain N content at harvest and increased NUT, NPFP, and NAE by 5.7%–40.1%, 2.5%–23.3%, and 3.9%–42.8%, respectively, at N63–N125. Therefore, PN applied using a single basal nitrogen fertilizer application method showed promising potential in maintaining a stable wheat yield and increasing N use efficiency with a 33% urea cut (approximately 63 kg N ha^–1^) compared to CN at the current wheat yield level on the NCP.

## Introduction

The global demand for food will rise by approximately 70% to feed 9.7–10.0 billion people by 2050 (Godfray et al., 2010). Wheat is now one of the most widely cultivated cereals, and nearly 670 million tons are produced annually, with developing regions accounting for approximately 53% and 50% of the total harvested area and yield, respectively (Shiferaw et al., 2013). Consequently, enhancing wheat production and productivity, especially in developing regions, is important for global food security.

N is an essential nutrient and one of the most yield-limiting factors for winter wheat (Hu et al., 2021). N applied before wheat planting is essential to establish shoot biomass and to develop tillers before the onset of winter dormancy (Sticksel et al., 1999; Kamiji et al., 2014); however, excessive N input could lead to ineffective tillers, increased lodging risk, and reduced wheat yield and quality accordingly (Mackown et al., 1989; Khan et al., 2020). N application could promote shoot elongation, leaf expansion, and chlorophyll synthesis, whereas N deficiency could lead to leaf senescence and chlorosis (Lv et al., 2021). Therefore, researchers and breeders typically regard leaf area duration (LAD) as a characteristic index reflecting wheat canopy characteristics (Wang et al., 2017b). Additionally, N could enhance the photosynthetic efficiency and promote the distribution and transfer of assimilates, resulting in a higher crop growth rate (CGR) (Ma et al., 2019). Previous studies have shown that N concentration is significantly positively correlated with CGR, which directly affects wheat grain filling, final yield, and the harvest index (HI) (Gaju et al., 2014).

Wheat yield has increased over the last century by 110 kg ha^–1^ per year, largely due to high N inputs (Austin, 1999). Increasing N application rates notably improve wheat grain yield; however, there was no yield benefit when N application rates exceeded 240 kg ha^–1^ on the North China Plain (NCP) (Si et al., 2020). Wang et al. (2017a) suggested that the optimal N fertilizer rate should be approximately 185 kg N ha^–1^ for wheat with an achieved yield of 7,000 kg ha^–1^ based on a literature review and field experiments in this region (Wang et al., 2017a). However, the abovementioned N application rates could not improve wheat yield alone and need to be combined with adaptive nitrogen management. N management practice in wheat is a challenging agronomic scenario due to its sensitivity to both under- and overapplication of N and the timing for tiller development and grain yield (Diacono et al., 2013; Shah et al., 2020; Huang et al., 2021). Currently, in most wheat production areas, farmers use basal nitrogen fertilizer combined with topdressing at the wheat jointing stage (Lu et al., 2015). However, there are two main issues in this type of N application method. First, it is difficult to quantify the amount of basal and topdressing fertilizer due to the variation in climate conditions, soil type, and even wheat cultivars. Second, the timing of topdressing depends largely on the irrigation condition and artificial input; however, for regions with no irrigation conditions, topdressing often occurs after rain and usually misses the best topdressing timing, resulting in fertilizer waste and even yield penalty. Therefore, coordination of wheat N demand with the innovation of N fertilizer and management practices was critically important to maximize wheat yield and nitrogen use efficiency.

Granular urea is the most common N source used for wheat production, and there are some new types of urea, such as environmentally friendly N and controlled-release urea, aiming to overcome the limitation of rapid volatilization and leaching of urea (Xu et al., 2013; Zheng et al., 2017). Controlled-release urea could prolong the N release period and improve wheat yield and N use efficiency with less N input. However, the cost of controlled-release urea is higher than that of granular urea, and coating materials for urea could lead to soil pollution as well, which are the main limiting factors for extensive promotion of controlled-release urea for field crops (Lawrencia et al., 2021). Ploy(aspartic acid) (PAA) is an eco-friendly and natural amino acid polymer that has a strong chelation, dispersion, and adsorption capacity for macro- and microelements and results in nutrient enrichment of the soil (Fang et al., 2006). Several previous studies demonstrated that PAA could enhance rice dry matter production, non-structural carbohydrates, yield, and nitrogen use efficiency in paddy fields (Deng et al., 2014, 2015, 2016). Wang et al. (2018) indicated that PAA promoted dry matter and N accumulation in the maize seedling stage (Wang et al., 2018). However, related knowledge regarding the effects of PAA-coated urea on wheat yield and nitrogen utilization, especially when combined with the one-time basal application method in clay loam soil, is still limited.

Therefore, in this study, we explored the effects of PN and CN on wheat yield and NUE at four N application rates with a one-time basal application method in clay loam soil on the NCP. The specific objectives of this study were to (1) evaluate the effects of CN and PN at different N rates on the mainly wheat agronomic traits (LAD, CGR, and DMA) and grain filling characteristics and (2) investigate the effects of PN on wheat yield and NUE with the one-time basal fertilizer application method.

## Materials and methods

### Experimental design

**FIGURE 1:**
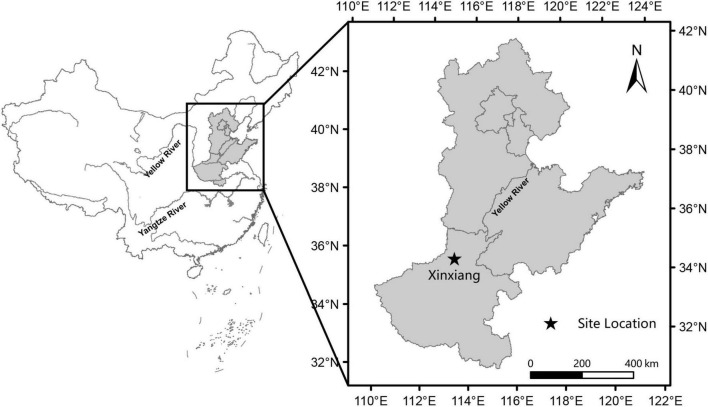
Location of the xinxiang experimental station and the North China Plain.

Long-term fertilizer experiments in clay loam soil were established at Xinxiang Experimental Station (35°16′N, 113°80′E) of the Institute of Crop Science, Chinese Academic of Agricultural Science (Figure 1) in 2015, and field data in the current study were collected from the 2017 to 2020 winter wheat growing seasons. The cropping system is a winter wheat-summer maize rotation system. Representative 0–40 cm topsoil samples were collected for selected chemical properties (Table 1).

**TABLE 1:** Characteristics of the 0–20 cm and 20–40 cm soil layers prior to wheat sowing during the three experimental years from 2018 to 2020.

Year	Soil layers	pH	Bulk density	Organic matter	Total N	Nitrate-N	Ammonium-N	Available P	Available K
	cm		g cm^3^	g kg^–1^	g kg^–1^	mg kg^–1^	mg kg^–1^	mg kg^–1^	mg kg^–1^
2018	0–20	7.9	1.3	13.4	1.2	15.5	3.8	15.9	113.5
	20–40	8.2	1.58	11.3	0.85	10.2	2.1	14.3	105.4
2019	0–20	8.1	1.27	12.5	1.1	13.8	3.1	16.1	109.9
	20–40	8.2	1.58	10.2	0.76	8.6	1.8	13.9	100.3
2020	0–20	8.1	1.28	13.1	1.1	12.9	2.3	15.8	100.2
	20–40	8.3	1.69	11.2	0.77	6.5	1.4	14.2	99.5

**FIGURE 2:**
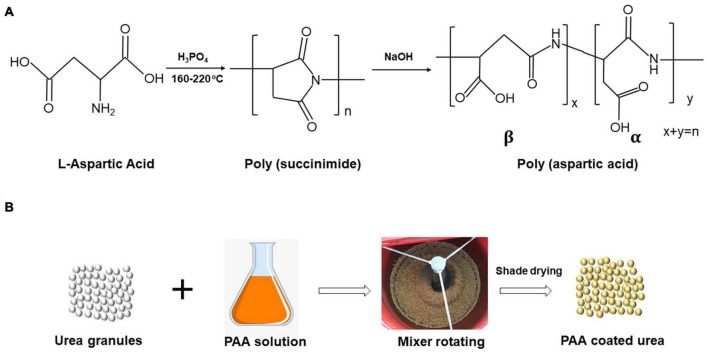
Synthesis of PAA **(A)** and preparation procedure of PAA-coated urea fertilizer **(B)**.

Plots were distributed in a randomized complete block design with four replicates. Treatments included two urea treatments, a control treatment (CN, uncoated urea) and poly(aspartic acid)-coated urea (PN). Both urea treatments consisted of four N rates: 0, 63, 125, and 188 kg N ha^–1^. PN was made in the laboratory in two steps, as shown in Figure 2 (Adelnia et al., 2021). First, a prepared poly(aspartic acid) (PAA) solution was mixed with poly(succinimide) powder [obtained by acid-catalyzed polycondensation of L-aspartic acid (CAS: 56-84-8)] with distilled water, and NaOH was added until the poly(succinimide) was completely dissolved (the reaction system formed a yellow clarified liquid) (Figure 2A). Second, urea was coated with PAA solution at 0.3% of the total urea rate and then air-dried naturally in the shade (Figure 2B).

The plot size was 70 m^2^ (7 m wide and 10 m long). In all treatments, Ca(H_2_PO_4_)_2_⋅H_2_O and K_2_SO_4_ were applied as basal fertilizers, providing 75 kg P ha^–1^ and 35 kg K ha^–1^, respectively. The wheat seed rate was approximately 240 kg ha^–1^ with a row spacing of 19 cm. Irrigation water was applied up to three times per plot: late October, early March, and late April. Herbicides were sprayed after planting and before emergence. Weeds in plots were removed by hand at the stem elongation stage. Pests and diseases were well controlled by the spraying of insecticide and fungicide at the stem elongation and anthesis stages. No obvious weed, pest, or disease stress was observed during the three-wheat experimental seasons.

### Meteorological data and phenological phases

**FIGURE 3:**
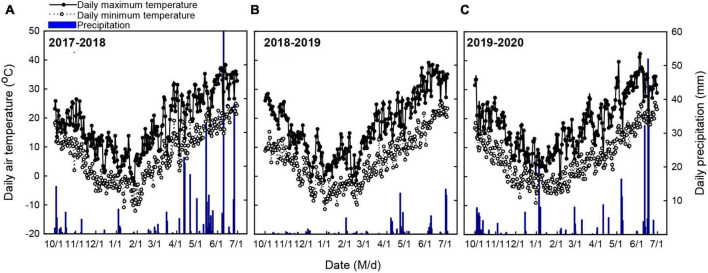
Daily maximum air temperature, daily minimum air temperature, and daily cumulative precipitation during the 2017–2018 **(A)**, 2018–2019 **(B)**, and 2019–2020 **(C)** winter wheat growing seasons.

Meteorological data, including daily air temperature (maximum, minimum, and average temperature), daily cumulative precipitation, and daily photosynthetically active radiation (PAR), were obtained from the local meteorological bureau (Figure 3). Phenological phases were recorded when more than 50% of the wheat in a plot was in a specific period according to the Zadoks staging system (Zadoks et al., 1974; Table 2).

**TABLE 2:** Phenological phases of winter wheat according to the Zadoks staging system during the three experimental years from 2017 to 2020.

Growth stages	Experimental year		
	
	2017–2018	2018–2019	2019–2020
Sowing	29-Oct	27-Oct	5-Nov
Seedling	8-Nov	7-Nov	13-Nov
Jointing	23-Mar	23-Mar	27-Mar
Heading	18-Apr	20-Apr	21-Apr
Anthesis	26-Apr	26-Apr	29-Apr
Ripening	2-Jun	5-Jun	2-Jun

### Agronomic traits

#### Leaf area and aboveground biomass accumulation

Six zones in the central rows of each plot were tagged at the wheat seedling stage, and each zone included approximately twenty consecutive plants. At the approximate jointing, heading, flowering, milk, and dough wheat growth stages, fifteen tagged plants were sampled and transported to the laboratory. The length and width of each green leaf were measured, and the leaf area (LA) was calculated by adding the individual LA (length x width x constant-coefficient, of which 0.5 for not yet fully expanded leaves and 0.75 for fully expanded leaves) and then multiplying by the plant density (plants m^–2^). After that, the samples were dried at 65°C to a constant weight and measured with a 0.01 g precision balance. The aboveground dry matter accumulation (DMA) was calculated as the plant biomass multiplied by the plant population at each growth stage. LAD and CGR were calculated by Eq. (1–2):


(1) $$\text{LAD}=\frac{\left[\left(L\,A_{2}+L\,A_{1}\right)\times \left(T_{2}-T_{1}\right)\right]}{2}$$

(2) $$\text{CGR}=\frac{\left(W\,2-W\,1\right)}{\left(T\,2-T\,1\right)}$$

where LA, W and T are the leaf area, dry matter accumulation and sample date, respectively.

### Leaf chlorophyll content

The latest expanded or flag leaf samples were collected from 10 plants in the inner row in each plot in the field, cleaned, and stored in liquid nitrogen for measurements in the laboratory. The leaf chlorophyll content (Chl *a* and Chl *b*) was measured according to the classical spectrophotometric method (Sarker et al., 2020). Briefly, using 95% ethanol was used to extract chlorophyll (each sample measured 0.2 g, and there were four replicates per sample), and then a UV-1800 spectrophotometer (Shimadzu, Kyoto, Japan) was used to measure the absorbance at 646 and 663 nm for Chl b and Chl a, respectively. Chlorophylls were calculated according to the Lambert-Beer law as mg g^–1^ FW.

### Grain filling characteristics

A total of 150 consecutive plants in the center rows were tagged at the anthesis stage, and sampling began 10 days after flowering. Twenty tagged plants were randomly sampled per plot every 5 days until the kernels reached physiological maturity. Kernels were dried at 65°C until constant weight and then measured with a 0.001 g balance. The grain filling characteristics were analyzed by the Richards Equation (Richards, 1959) in Eq. (3):


(3) y=a(1+e⁢^⁢(b-c⁢x))⁢^⁢(1/d)

where y is the wheat kernel weight (mg); x is the days after anthesis; a is the final kernel weight (mg); and b, c, and d are the equation parameters.

Tmax represents the days needed (post-anthesis) to reaching the maximum grain-filling rate (Gmax) and is calculated by Eq. (4):


(4) $$T\,m\,a\,x=\frac{\left(b-l\,n\,d\right)}{c}$$

Wmax represents the kernel weight at the maximum grain-filling rate, calculated by Eq. (5):


(5) W⁢m⁢a⁢x=a×(d+1)⁢^⁢(-1/d)

Gmax represents the maximum grain-filling rate, calculated by Eq. (6):


(6) G⁢m⁢a⁢x=((c×W⁢m⁢a⁢x)/d)×(1-(W⁢m⁢a⁢x/a)×d)

Gmean represents the average grain-filling rate and is calculated by Eq. (7):


(7) Gmean=a×c/2×(d+2)

P represents the active grain-filling period and is calculated by Eq. (8):


(8) P=2×(d+2)/c

### Yield and yield components

At harvest, 1.9 m^2^ in the inner five rows (0.95 m width and 2 m length) of each plot was harvested manually to determine the wheat yield and yield components. The spike number was measured as the number of effective spikes divided by the sampled area. Three separate 1,000-kernel samples were randomly counted in each plot with a weight error less than 0.5 g among the three samples, and then the kernel weight was determined after over-drying at 60°C until constant weight. Kernel moisture was measured by an M-8188A grain moisture analyzer (KETT, Tokyo, Japan), 10 times per sample. The grains from each plot were weighed and corrected to a water content of 13.5% for the final grain yield.

### Stover N content and grain N content

Samples were collected from each harvested plot, threshed to manually separate the stover and grain, and then dried at 60°C to constant weight. Stover and grain N nitrogen contents were measured by an SPD50 according to the Kjeldahl method (Kjeldahl Azotometer, Beijing, China) (Deng et al., 2019). Then, total N uptake (NUT), N partial factor productivity (NPFP), N recovery efficiency (NRE), and N agronomic efficiency (NAE) were calculated by Eq. (9–12):


(9) $$\text{NUT}\left(\text{kg}\;{\text{ha}}^{\text{-1}}\right)=Biomass_{\text{stover}}\times NC_{\text{stover}}+Grain\times NC_{\text{grain}}$$

(10) $$\text{NPFP}\,\left(\mathrm{kg}\,{\text{kg}}^{-1}\right)=\frac{G\,Y}{N}$$

(11) $$\mathrm{NRE}\left(\%\right)=\frac{\left(N\,U\,T-N\,U\,T_{0}\right)}{N}$$

(12) $$\mathrm{NAE}\,\left(\mathrm{kg}\,{\text{kg}}^{-1}\right)=\frac{\left(G\,Y-G\,Y_{0}\right)}{N}$$

where NC_*stover*_ and NC_*grain*_ are the respective stover (without the kernel) and grain N contents at harvest, respectively. GY and GY_0_ are the grain yield at maturity with and without N input, respectively. NUT_0_ is the total N uptake by control (unfertilized) plants, and N represents the nitrogen fertilizer rate.

### Statistical analysis

The effect of treatment (nitrogen, PAA) on the wheat grain yield and yield components was analyzed by the General Linear Model procedure (GLM) in SPSS 20.0 (SPSS Inc., Chicago, United States), where means were compared using the least significant differences test (LSD) at the 5% (*p* ≤ 0.05) probability level. The curves of the grain filling dynamics were analyzed by CurveExpert Pro (Hyams Development) according to the Richards Equation (Richards, 1959). Figures were generated in R 4.0.5 (R Core Team, 2021).

## Results

### Growing conditions

Figures 3A–C shows the daily maximum air temperature (Tmax), minimum air temperature (Tmin), and cumulative precipitation at the Xinxiang experimental station during 2017–2020. During the whole wheat growing cycle, Tmax, Tmin, and total cumulative precipitation were 15.7°C, 4.7°C, and 190.6 mm in 2017–2018, 14.9°C, 3.5°C, and 57.2 mm in 2018–2019, and 16.1°C, 4.0°C, and 114.1 mm in 2019–2020. During the vegetative growth period (sowing-jointing), the average Tmin and total precipitation were 0.2°C and 35.7 mm in 2017–2018, –0.6°C and 16.3 mm in 2018–2019, and 0.5°C and 65.4 mm in 2019–2020. During the reproductive growth period (jointing-ripening), the daily air temperature was similar (25.3 ± 0.5°C) during the three experimental years. However, there was a considerable difference in cumulative precipitation among the three experimental seasons (154.9, 40.9, and 48.7 mm in 2017–2018, 2018–2019, and 2019–2020, respectively).

### Yield and yield components

The year, N rates, and treatments had significant (*p* < 0.05) effects on the wheat yield. As shown in Table 3, wheat yield across all treatments in 2019–2020 (7.7 Mg ha^–1^) was higher than that in 2018–2019 (7.4 Mg ha^–1^) and 2017–2018 (6.8 Mg ha^–1^). N rates increased wheat yield significantly; in comparison with 63 kg N ha^–1^ (N63), wheat yield under 125 kg N ha^–1^ (N125) and 188 kg N ha^–1^ (N188) increased by 48.8% and 58.0%, respectively, across the three experimental seasons. PN increased wheat yield by 9.6%, 9.2%, and 2.2% in comparison with CN under N63, N125, and N188, respectively, across the three experimental years.

**TABLE 3:** Wheat yield, yield components (kernel number, kernel weight, and spike number), and harvest index at four N rates under the control (CN) and poly(aspartic acid)-coated urea (PN) treatments from 2017 to 2020.

Year	Nitrogen rate	Treatment	Yield	Kernel number	Kernel weight	Spike number	Harvest index
							
	kg ha^–1^		kg ha^–1^	Spike^–1^	mg kernel^–1^	× 10^4^ ha^–1^	
2017–2018	63	CN	5963.6 c^§^	30.6 a	42.4 a	529.4 c	0.50 b
		PN	6114.0 c	31.2 a	43.4 a	542.5 bc	0.51 b
	125	CN	6492.3 bc	32.3 a	41.5 ab	550.9 bc	0.52 a
		PN	6965.9 b	32.0 a	42.0 ab	560.5 bc	0.52 a
	188	CN	7662.7 a	31.4 a	40.4 b	602.6 a	0.51 ab
		PN	7504.5 a	31.9 a	40.3 b	582.0 ab	0.51 ab
2018–2019	63	CN	4614.4 c	24.4 d	44.6 b	414.9 c	0.43 c
		PN	5242.2 c	28.8 c	44.9 ab	439.5 bc	0.44 c
	125	CN	7961.6 b	31.8 b	45.5 ab	503.1 b	0.49 ab
		PN	9201.1 a	37.6 a	45.8 a	591.7 a	0.51 a
	188	CN	8412.6 ab	30.5 bc	45.9 a	578.5 a	0.50 ab
		PN	8992.1 a	36.4 a	44.9 ab	581.6 a	0.49 ab
2019–2020	63	CN	4789.8 d	25.2 c	43.7 ab	538.6 b	0.38 d
		PN	5494.4 c	26.5 c	44.5 a	526.8 b	0.41 c
	125	CN	8454.6 b	32.4 b	44.1 ab	660.1 a	0.45 ab
		PN	8851.8 a	35.7 a	44.2 ab	664.0 a	0.48 a
	188	CN	9101.5 a	33.5 b	43.3 ab	636.8 a	0.45 ab
		PN	9233.0 a	33.7 b	43.0 b	649.6 a	0.47 a
**ANOVA**							
Year			***	ns^¶^	***	***	***
Nitrogen rate			***	***	**	***	***
Treatment			***	***	ns	ns	**

^§^The LSD at p ≤ 0.05 is used to compare the treatment means within the same year; means within the same year followed by the same letter are not significantly different.

^¶^ns: Not significant (p > 0.05); “**” and “***” represent significant difference at the 0.01 and 0.001 probability levels, respectively.

As shown in Table 3, N rates and treatment showed significant (*p* < 0.05) effects on kernel number and spike number. The kernel numbers under N125 and N188 were 21.1% and 18.4% higher than those under N63. PN significantly increased the kernel number per spike by 7.8%, 9.2%, and 7.0% under N63, N125, and N188, respectively, across the three experimental years. Similarly, the spike number increased by 18.0% and 21.4% under N125 and N188, respectively, in comparison with N63. PN significantly increased the spike number by 6.0% under N125 but showed no significant difference under N63 and N188.

Kernel weight differed significantly among experimental years (Table 3). The kernel weights in 2019–2020 and 2018–2019 were 5.1% and 8.6% higher, respectively, than those in 2017–2018 among the N rates and treatments. N rates and treatments showed no significant effect on the kernel weight. However, the kernel weight showed a decreasing tendency with increasing nitrogen application rate during the experimental years.

### Leaf area duration, leaf chlorophyll content, and crop growth rate

During the jointing-anthesis growth stage, the leaf area duration (LAD), leaf chlorophyll content (Chl), and crop growth rate (CGR) differed significantly among years and N rates, while PN showed no significant effect on these traits (Table 4). The average LAD was 39.4% higher in 2018–2019 and 14.5% higher in 2019–2020 than in 2017–2018. The LAD under N125 and N188 was 45.5% and 50.0% higher, respectively, than that under N63 across the 3 years. In comparison to N63, Chl and CGR under N188 and N125 increased by 15.8%–20.7% and 5.0%–11.5%, respectively (Table 4).

**TABLE 4:** The main agronomic traits of wheat during the jointing-anthesis and anthesis-ripening growing stages at four N rates under the control (CN) and poly(aspartic acid)-coated urea (PN) treatments from 2017 to 2020.

Year	Nitrogen rate	Treatment	Growth stages					
			
			Jointing-anthesis			Anthesis-ripening		
						
			LAD	Chl	CGR	LAD	Chl	CGR
								
	kg ha^–1^		m^2^ m^–2^ d^–1^	mg g^–1^	g m^–2^ d^–1^	m^2^ m^–2^ d^–1^	mg g^–1^	g m^–2^ d^–1^
2017–2018	0	CN	30.4 d^§^	3.1 d	8.4 e	33.3 e	2.0 e	5.3 d
		PN	–	–	–	–	–	–
	63	CN	71.8 c	3.5 c	21.7 abc	86.9 d	3.7 cd	10.4 c
		PN	72.5 c	3.8 b	20.5 bcd	95.7 c	3.6 d	15.4 b
	125	CN	82.0 b	3.6 bc	19.6 cd	117.8 a	3.9 bcd	13.9 bc
		PN	86.0 b	3.6 bc	17.7 d	121.5 a	3.8 bc	20.0 a
	188	CN	91.1 a	4.3 a	24.3 a	111.9 b	4.0 b	14.6 b
		PN	86.1 b	3.7 b	23.0 ab	119.8 a	4.2 a	16.0 b
2018–2019	0	CN	41.1 c	2.7 c	5.8 d	34.0 f	2.3 f	5.3 c
		PN	33.6 c	2.4 d	5.8 d	30.9 f	2.6 e	5.1 c
	63	CN	85.9 b	2.7 c	16.6 c	56.0 e	2.8 de	8.2 c
		PN	90.3 b	2.7 c	16.2 c	72.5 d	3.0 d	13.7 b
	125	CN	125.1 a	3.3 b	18.1 bc	119.5 c	3.9 c	14.9 b
		PN	113.4 ab	3.5 a	19.1 b	152.1 a	4.3 a	17.5 ab
	188	CN	140.3 a	3.2 b	17.1 bc	140.4 b	4.0 bc	19.4 a
		PN	127.2 a	3.3 b	22.0 a	151.0 a	4.2 ab	19.6 a
2019–2020	0	CN	25.6 c	1.1 d	6.6 c	24.8 c	1.0 d	7.6 d
		PN	25.9 c	1.1 d	6.5 c	28.2 c	1.0 d	7.3 d
	63	CN	65.5 b	1.9 c	19.5 ab	45.2 b	1.6 c	7.4 d
		PN	56.0 b	1.9 c	17.7 ab	50.8 b	1.5 c	13.6 c
	125	CN	113.6 a	2.5 a	20.8 ab	147.2 a	3.2 a	12.4 c
		PN	110.7 a	2.1 b	21.2 a	130.3 a	2.5 b	15.1 bc
	188	CN	107.9 a	2.6 a	20.9 ab	132.3 a	3.1 a	18.3 ab
		PN	106.7 a	2.4 a	17.4 b	135.9 a	3.2 a	19.6 a
**ANOVA**								
Year			***	***	***	**	***	ns
Nitrogen rate			***	***	**	***	***	***
Treatment			ns^¶^	ns	ns	***	ns	***

LAD, leaf area duration; Chl, leaf chlorophyll content; CGR, crop growth rate.

^§^The LSD at p ≤ 0.05 is used to compare the treatment means within the same year; means within the same year followed by the same letter are not significantly different.

^¶^ns: Not significant (p > 0.05); “**” and “***” represent significant difference at the 0.01 and 0.001 probability levels, respectively.

During the anthesis-ripening growth stage, the average LAD and Chl were 1.9% and 55.1% higher in 2017–2018 and 7.8% and 48.1% higher in 2018–2019 than in 2019–2020. LAD, Chl, and CGR increased significantly with increasing N rates during this period. Compared with N63, the LAD, Chl, and CGR increased by 93.6%, 33.5%, and 36.3% under N125 and by 94.3%, 40.0%, and 56.3% under N188. PN significantly increased LAD and CGR by 16.4% and 64.3% under N63, 5.1% and 27.5% under N125, and 5.8% and 5.4% under N188 across the three experimental years.

### Dry matter accumulation

**FIGURE 4:**
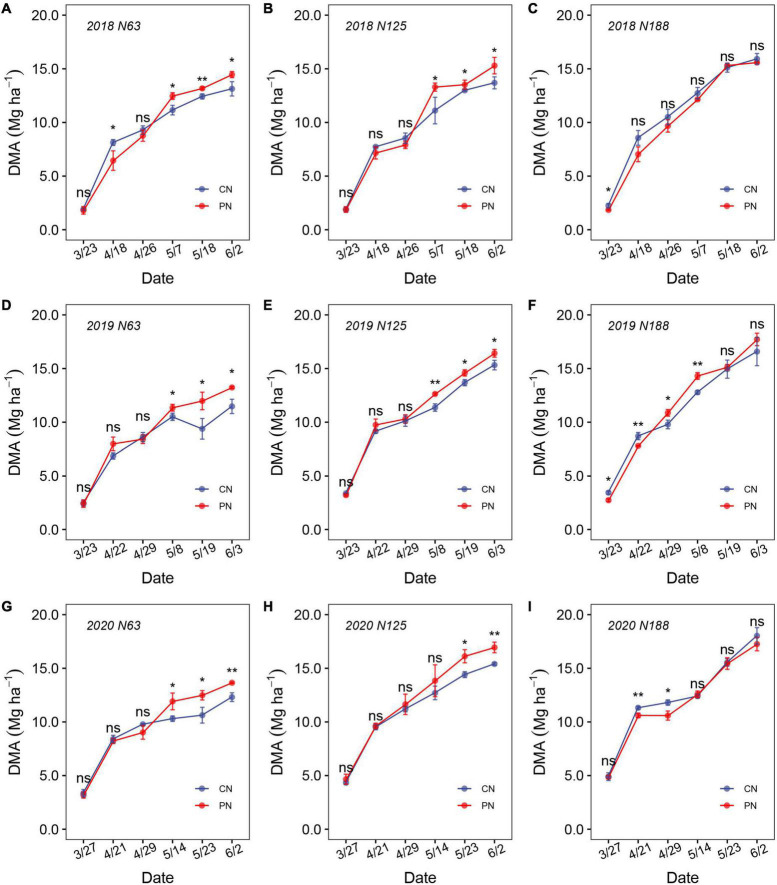
Dynamic variation in biomass accumulation from the jointing to ripening stages at N63 **(A,D,G)**, N125 **(B,E,H)**, and N188 **(C,F,I)** under the control (CN) and poly(aspartic acid)-coated urea (PN) treatments during the 2017–2018 **(A–C)**, 2018–2019 **(D–F)** and 2019–2020 **(G–I)** growing seasons. (ns means not significant, “*” and “**” above the columns indicate significance at the *p* = 0.05 and *p* = 0.01 levels, respectively, within a group).

N rates and PN both showed significant effects on DMA across the three experimental years (Figures 4A–I). The maximum DMA was observed under N188, which was 29.6% and 8.6% higher than that under N63 and N125, respectively. The effect of PN on DMA depended more on the wheat growth stages and N rates. Before anthesis, PN decreased DMA at the same N rate, but there was no significant difference for most parts. In contrast, after anthesis, PN significantly increased DMA by 13.7% and 10.1% under N63 and N125, respectively, whereas no significant difference was detected under N188 (Figure 4).

### Grain-filling characteristics

**FIGURE 5:**
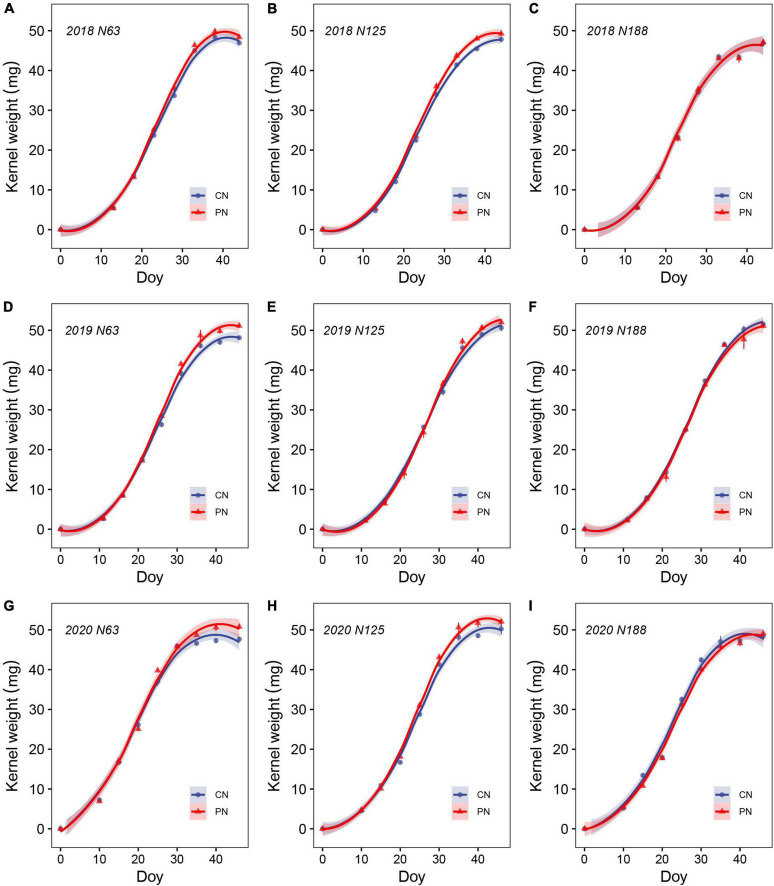
Wheat kernel dry weight dynamics at N63 **(A,D,G)**, N125 **(B,E,H)**, and N188 **(C,F,I)** in the control (CN) and poly(aspartic acid)-coated urea (PN) treatments during the 2017–2018 **(A–C)**, 2018–2019 **(D–F)** and 2019–2020 **(G–I)** growing seasons.

The year, N rates, and treatments showed significant effects on the wheat grain-filling characteristics (Figures 5A–I and Table 5). The time required for kernels to reach the maximum grain-filling rate (Tmax) under N125 and N188 was 7.2 and 6.0% longer than that under N63, respectively. In comparison with N63, the Gmax under N125 and N188 decreased by 6.8% and 2.5%, respectively. The kernel weight at the maximum grain-filling rate (Wmax) and the active grain-filling period (P) showed no apparent difference among the N rates. As shown in Figure 5, PN increased the kernel weight, especially under N63 and N125, and the Gmax and Wmax under N125 in the PN treatment were 21.7% and 6.7% higher, respectively, compared to those in CN.

**TABLE 5:** Grain-filling characteristics of wheat kernels at three N rates under the control (CN) and poly(aspartic acid)-coated urea (PN) treatments from 2017 to 2020.

Year	Nitrogen rate	Treatment	Richard Model	R^2^	Tmax	Wmax	Gmax	P
				
	kg ha^–1^				d	—mg kernel^–1^—		d
2017–2018	63	CN	*y* = 48.4/[1+exp(8.4–0.3x)^0.4]	0.9972	25.4	28.8	2.8	29.0
		PN	*y* = 50.1/[1+exp(6.6–0.3x)^0.6]	0.9978	24.1	27.4	2.9	28.2
	125	CN	*y* = 49.0/[1+exp(3.7–0.2x)^1.4]	0.9999	23.1	23.0	1.9	31.0
		PN	*y* = 50.2/[1+exp(5.0–0.2x)^0.9]	0.9997	23.7	25.6	2.6	30.1
	188	CN	*y* = 46.5/[1+exp(6.1–0.2x)^0.7]	0.9980	23.7	25.2	2.6	28.8
		PN	*y* = 46.3/[1+exp(6.2–0.2x)^0.7]	0.9979	23.7	25.1	2.6	28.4
2018–2019	63	CN	*y* = 48.5/[1+exp(7.1–0.3x)^0.6]	0.9984	25.8	27.2	2.7	29.5
		PN	*y* = 51.4/[1+exp(6.9–0.3x)^0.6]	0.9994	25.6	28.2	2.9	28.4
	125	CN	*y* = 53.2/[1+exp(4.1–0.2x)^1.2]	0.9989	26.1	25.6	2.1	34.5
		PN	*y* = 53.1/[1+exp(6.5–0.2x)^0.7]	0.9995	27.5	28.5	2.8	30.5
	188	CN	*y* = 52.5/[1+exp(6.5–0.2x)^0.7]	0.9996	27.3	28.5	2.7	31.2
		PN	*y* = 51.3/[1+exp(6.2–0.2x)^0.7]	0.9989	27.0	27.2	2.7	30.6
2019–2020	63	CN	*y* = 47.8/[1+exp(6.5–0.3x)^0.5]	0.9984	20.7	28.0	2.6	29.8
		PN	*y* = 52.2/[1+exp(3.8–0.2x)^0.9]	0.9975	19.8	26.4	2.5	32.1
	125	CN	*y* = 49.7/[1+exp(10.2–0.3x)^0.3]	0.9992	26.0	31.4	2.9	28.8
		PN	*y* = 52.4/[1+exp(8.4–0.3x)^0.4]	0.9998	25.1	31.3	3.0	28.7
	188	CN	*y* = 48.0/[1+exp(10.0–0.4x)^0.3]	0.9976	24.5	30.6	2.8	28.7
		PN	*y* = 48.5/[1+exp(6.3–0.2x)^0.6]	0.9990	23.7	27.3	2.6	31.0

Tmax, days needed for reaching the maximum grain-filling rate; Wmax, kernel weight (mg) at the maximum grain-filling rate; Gmax, maximum grain-filling rate; P, active grain-filling period.

### Nitrogen utilization characteristics

As shown in Table 6, the average wheat stover and grain N contents in 2017–2018 were 0.54% and 2.16% on average, respectively, which were higher than those in 2018–2019 (0.33% and 1.86%) and 2019–2020 (0.47% and 2.02%). The stove N content and grain N content increased with increasing N rates. Compared with N63, the stove N content and grain N content under N125 and N188 increased by 52.3%–71.2% and 17.5%–18.8%, respectively. PN increased the stover N content and grain N contents by an average of 12.4% and 2.9% on average, respectively, across the three N rates.

**TABLE 6:** Wheat stover and grain N content, total nitrogen uptake (NUT), nitrogen partial factor productivity (NPFP), and nitrogen agronomic efficiency (NAE) at three N rates under the control (CN) and poly(aspartic acid)-coated urea (PN) treatments from 2017 to 2020.

Year	Nitrogen rate	Treatment	Stover N content	Grain N content	NUT	NPFP	NRE	NAE
				
	kg ha^–1^		%	%	kg ha^–1^	kg kg^–1^	%	kg kg^–1^
2017–2018	63	CN	0.36 c^§^	2.18 a	136.6 d	95.3 a	64.7 a	60.9 a
		PN	0.47 c	2.15 ab	145.7 cd	97.8 a	71.3 a	63.4 a
	125	CN	0.57 b	2.13 b	158.4 c	52. b	40.4 b	34.7 b
		PN	0.58 b	2.19 a	174.8 b	55.8 b	46.4 b	38.5 b
	188	CN	0.55 b	2.14 b	188.0 a	40.9 c	34.2 c	29.4 c
		PN	0.70 a	2.17 ab	197.6 a	40.0 c	36.5 c	28.6 c
2018–2019	63	CN	0.14 c	1.57 c	72.1 c	73.8 ab	24.6 d	35.8 bc
		PN	0.25 c	1.39 c	81.7 c	83.8 a	26.2 d	47.8 a
	125	CN	0.38 b	1.97 b	171.3 b	63.7 b	45.1 b	44.7 ab
		PN	0.35 b	1.98 b	193.9 ab	73.6 ab	54.4 a	55.6 a
	188	CN	0.41 a	2.09 a	191.9 ab	44.9 c	35.1 c	32.2 c
		PN	0.47 a	2.14 a	216.4 a	48.0 c	41.8 b	35.9 bc
2019–2020	63	CN	0.34 c	1.56 c	86.5 e	71.3 b	27.4 d	36.9 c
		PN	0.35 c	1.93 c	121.1 d	87.8 a	52.0 b	52.7 a
	125	CN	0.54 b	2.14 b	211.9 c	65.8 c	59.9 a	48.6 b
		PN	0.49 b	2.25 a	224.0 b	70.8 b	63.9 a	53.2 a
	188	CN	0.52 b	2.08 b	227.6 b	48.5 d	43.8 c	37.1 c
		PN	0.61 a	2.18 a	245.2 a	49.2 d	47.8 b	37.5 c
**ANOVA**								
Year			*	*	***	ns^¶^	*	ns
Nitrogen rate			***	***	***	***	***	***
Treatment			***	*	***	***	***	***

NUT, total nitrogen uptake; NPFP, nitrogen partial factor productivity; NRE, N recovery efficiency; NAE, nitrogen agronomic efficiency.

^§^The LSD at p ≤ 0.05 is used to compare the treatment means within the same year; means within the same year followed by the same letter are not significantly different.

^¶^ns, Not significant (p > 0.05); “*” and “***” represent significant difference at the 0.05 and 0.001 probability levels, respectively.

The total nitrogen uptake (NUT) at harvest in 2019–2020 was 11.5% and 20.4% higher than that in 2017–2018 and 2018–2019, respectively. N rates increased NUT but sharply decreased N partial factor productivity (NPFP), N recovery efficiency (NRE), and N agronomic efficiency (NAE). The NUT under N188 was 96.8% and 11.7% higher than that under N63 and N125, respectively, while the NPFP, NRE, and NAE under N188 were 46.7%, 10.2%, and 32.5%, 28.9%, 22.9%, and 27.1% lower than those under N63 and N125, respectively. PN increased NUT, NPFP, NRE, and NAE by 10.8%, 9.1%, 17.4%, and 16.7%, respectively, compared to CN. It is noteworthy that PN significantly increased NUT, NPFP, NRE, and NAE mainly under N63 and N125, while no significant difference was observed under N188.

## Discussion

The present study was set up to assess the effect of poly(aspartic acid) (PAA)-coated urea (PN) on winter wheat yield and N use efficiency. As mentioned in the literature review, N is currently one of the most yield-limiting factors for winter wheat production (Yue et al., 2012). Additionally, wheat N management has long been considered a challenging agronomic scenario (Biermacher et al., 2006; Liu et al., 2020b). The effect of N application methods on wheat yield varied notably with considerable changes in temperature and precipitation during the wheat growing season (Hu et al., 2019). In the present study, cloudy and rainy weather during the grain filling period could partly explain the yield penalty in the 2017–2018 season. Overall, wheat yield increased with increasing N rates, i.e., under 188 kg N ha^–1^ with the one-time basal application method, but was lower than that in previous studies in this region (Cui et al., 2008, 2010; Xu et al., 2018). PN showed a significant advantage in increasing wheat yield under N63 and N125; however, there was no significant difference under N188 in the current study. This might be due to the limitation of wheat variety N use efficiency or yield potential under high N availability conditions. Wang et al. (2011) pointed out that the potential yield of Jimai 22 was approximately 9.0 Mg ha^–1^, and available nitrogen in the plow layer under 188 kg N ha^−1^ was sufficient for winter wheat during the whole growing season on the NCP (Wang et al., 2011).

One very important issue for the N fertilizer one-time basal application method is that excessive N fertilizer promotes tiller development in the wintering period (typically lasting 2–4 weeks on the NCP) and increases the rates of ineffective tillers (Ma et al., 2018; Zhang et al., 2020). In this study, the spike number increased with increasing N rates, while PN increased the spike number at harvest only under N125 and showed no significant effect under N63 and N188. Another important issue for the N fertilizer one-time basal application method is that there will not be sufficient available N left in the top layer of soil during the later growing stage of wheat (Jensen et al., 2000), and the kernel number and kernel weight will decrease accordingly (Demotes-Mainard and Jeuffroy, 2001). In the present study, the kernel number per spike increased with increasing N rates, and PN showed a similar trend in promoting the kernel number. The N rates increased the kernel weight but depended largely on the weather conditions. For example, the nitrogen rate had little effect on the kernel weight under continuous rainy conditions during the wheat grain-filling stage (Pandey et al., 2001).

N input could increase the leaf chlorophyll content, LAD, and therefore crop growth rate. In the current study, the LAD, chlorophyll content, and CGR increased with increasing nitrogen rates. PN increased LAD and CGR during the grain-filling stage but showed no effect on the leaf chlorophyll content. It is speculated that PN results in a slow release effect on nitrogen fertilizer since the leaf chlorophyll content is directly related to the leaf nitrogen content (Shadchina and Dmitrieva, 1995), and PN promotes wheat nitrogen absorption, and the chlorophyll content will increase accordingly. Nitrogen application can increase biomass accumulation (Salvagiotti and Miralles, 2008). However, the present study showed that PN had no significant effect on improving DMA compared to CN during the jointing to anthesis growth stages. It is surprising is that PN significantly increased DMA during the entire grain-filling stage compared to CN. During the grain-filling stage, excessive nitrogen application could prolong the entire grain-filling period rather than the effective grain-filling period and decrease the Gmax (Liu et al., 2020a). In the current study, PN increased the wheat Gmax and kernel weight at the Gmax under N63 and N125 compared to CN.

N applied with the one-time basal application method could increase the leaf chlorophyll content, LAD, and CGR, which combined increase DMA before anthesis and promote the formation of effective spikes and kernel number per spike (Shadchina and Dmitrieva, 1995; Gaju et al., 2014). However, excessive nitrogen application in the early growing stage of wheat can result in luxury consumption and negatively effect DMA (Wang et al., 2017a). DMA during the postanthesis stage increased the formation of kernel number per spike and increase the harvest index. Overall, PN prevented luxury consumption in wheat before anthesis, and the available N retained in the top layer of soil during the later wheat growing stage enhanced DMA and grain filling.

Several previous studies have shown the potential of controlled-release urea in increasing plant N absorption and utilization, and N inputs have been found to significantly increase the crop N content and total nitrogen uptake (Gao et al., 2021; Jiang et al., 2021). There are currently two explanations concerning the mechanism by which PN promoted nitrogen utilization in this study. Poly(aspartic acid) is a hydrophilic and biodegradable polymer of aspartic acid with good dispersibility and adsorption capacity (Vega-Chacon et al., 2017). Deng et al. (2014) found that PN could reduce N loss and increase rice N uptake and N use efficiency in paddy soil (Deng et al., 2014). However, Wang et al. (2018) found that PN could increase maize nitrogen assimilation by enhancing nitrate reductase activity in a scarce NO_3_^–^ environment (Wang et al., 2018). In the present study, PN increased maize plant nitrogen uptake and nitrogen utilization efficiency under low and medium nitrogen rates (N63 and N125) but seldom showed a significant advantage under N188 compared to CN. These results indicated that PN promoted wheat nitrogen utilization, which was mainly attributed to the slow release of the N in clay loam soil, and enhanced N supply to meet plant N requirements during the later wheat growing stage. However, there are still many unanswered questions, such as the mechanisms and effects of PN on nutrient release and nutrient absorption, which need to be further investigated.

## Conclusion

N management is one of the major limiting factors in increasing farm profitability. The current study investigated the effect of the eco-friendly material poly(aspartic acid)-coated urea (PN) on wheat yield and N use efficiency. Our key findings are as follows: (1) in comparison with CN, PN increased wheat yield significantly under N63 and N125, while no obvious effect on wheat yield was found under N188 compared to CN. The increase in wheat yield induced by PN was mainly attributed to the increasing spike number and kernel number, especially the kernel number. (2) PN increased the leaf area duration (LAD) and crop growth rate (CGR), which together enhanced the dry matter accumulation (DMA) during the grain filling stage, and (3) PN increased the total N uptake (NUT), N partial factor productivity (NPFP), N recovery efficiency (NRE) and N agronomic efficiency (NAE) compared to CN under N63 and N125 but showed no significant effect under N188. Consequently, the use of moderate amounts (125 kg N ha^–1^) of PN showed great potential to reduce wheat N input by approximately 33% (compared to 188 kg N ha^–1^ CN) without a yield penalty at the current yield level with the one-time basal application method in clay loam soil on the NCP.

## Data availability statement

The raw data supporting the conclusions of this article will be made available by the authors, without undue reservation.

## Author contributions

ZD designed and guided this research. PY, XD, LL, ZM, and JD organized the design and conducted the field experiments. MF carried out the lab analysis. PY analyzed the data and wrote the manuscript. All authors contributed to the article and approved the submitted version.

## Abbreviations

PN: poly(aspartic acid) coated urea
CN: control treatment
LAD: leaf area duration
CGR: crop growth rate
DMA: dry matter accumulation
Tmax: days needed for reaching the maximum grain-filling rate
Wmax: kernel weight at the maximum grain-filling rate
Gmax: maximum grain-filling rate
Gmean: average grain-filling rate
P: active grain-filling period
NUT: total N uptake
NPFP: N partial factor productivity
NRE: N recovery efficiency
NAE: N agronomic efficiency.

## References

[B1] Adelnia H. Tran H. D. N. Little P. J. Blakey I. Ta H. T. (2021). Poly(aspartic acid) in biomedical applications: From polymerization, modification, properties, degradation, and biocompatibility to applications. *ACS Biomater. Sci. Eng.* 7 2083–2105. 10.1021/acsbiomaterials.1c00150 33797239

[B2] Austin R. B. (1999). Yield of wheat in the United Kingdom: Recent advances and prospects. *Crop Sci.* 39 1604–1610. 10.2135/cropsci1999.3961604x

[B3] Biermacher J. T. Epplin F. M. Brorsen B. W. Solie J. B. Raun W. R. (2006). Maximum benefit of a precise nitrogen application system for wheat. *Precis. Agric.* 7 193–204. 10.1007/s11119-006-9017-6

[B4] Cui Z. L. Zhang F. S. Chen X. P. Dou Z. X. Li J. L. (2010). In-season nitrogen management strategy for winter wheat: Maximizing yields, minimizing environmental impact in an over-fertilization context. *Field Crops Res.* 116 140–146. 10.1016/j.fcr.2009.12.004

[B5] Cui Z. L. Zhang F. S. Chen X. P. Miao Y. X. Li J. L. Shi L. W. (2008). On-farm evaluation of an in-season nitrogen management strategy based on soil N-min test. *Field Crops Res.* 105 48–55. 10.1016/j.fcr.2007.07.008

[B6] Demotes-Mainard S. Jeuffroy M. H. (2001). Incorporating radiation and nitrogen nutrition into a model of kernel number in wheat. *Crop Sci.* 41 415–423. 10.2135/cropsci2001.412415x

[B7] Deng A. X. Zhang X. Zhang X. Y. Qian H. Y. Zhang Y. Chen C. L. (2019). Impacts of wheat photosynthate allocation on soil N2O emission during post-anthesis period. *Biol. Fertil. Soils* 55 643–648. 10.1007/s00374-019-01377-4

[B8] Deng F. Wang L. Mei X. F. Li S. X. Pu S. L. Ren W. J. (2016). Polyaspartate Urea and Nitrogen Management Affect Nonstructural Carbohydrates and Yield of Rice. *Crop Sci.* 56 3272–3285. 10.2135/cropsci2016.02.0130

[B9] Deng F. Wang L. Ren W. J. Mei X. F. (2014). Enhancing nitrogen utilization and soil nitrogen balance in paddy fields by optimizing nitrogen management and using polyaspartic acid urea. *Field Crops Res.* 169 30–38. 10.1016/j.fcr.2014.08.015

[B10] Deng F. Wang L. Ren W. J. Mei X. F. Li S. X. (2015). Optimized nitrogen managements and polyaspartic acid urea improved dry matter production and yield of indica hybrid rice. *Soil Tillage Res.* 145 1–9. 10.1016/j.still.2014.08.004

[B11] Diacono M. Rubino P. Montemurro F. (2013). Precision nitrogen management of wheat. A review. *Agron. Sustain. Dev.* 33 219–241. 10.1007/s13593-012-0111-z

[B12] Fang L. Zhao Y. Tan T. W. (2006). Preparation and water absorbent behavior of superabsorbent polyaspartic acid resin. *J. Polym. Res.* 13 145–152. 10.1007/s10965-005-9022-x

[B13] Gaju O. Allard V. Martre P. Le Gouis J. Moreau D. Bogard M. (2014). Nitrogen partitioning and remobilization in relation to leaf senescence, grain yield and grain nitrogen concentration in wheat cultivars. *Field Crops Res.* 155 213–223. 10.1016/j.fcr.2020.107778 32549650 PMC7182295

[B14] Gao Y. X. Song X. Liu K. X. Li T. G. Zheng W. K. Wang Y. (2021). Mixture of controlled-release and conventional urea fertilizer application changed soil aggregate stability, humic acid molecular composition, and maize nitrogen uptake. *Sci. Total Environ.* 789:147778. 10.1016/j.scitotenv.2021.147778 34051498

[B15] Godfray H. C. J. Beddington J. R. Crute I. R. Haddad L. Lawrence D. Muir J. F. (2010). Food Security: The Challenge of Feeding 9 Billion People. *Science* 327 812–818. 10.1126/science.1185383 20110467

[B16] Hu C. L. Sadras V. O. Lu G. Y. Jin X. Xu J. X. Ye Y. L. (2019). Dual-purpose winter wheat: Interactions between crop management, availability of nitrogen and weather conditions. *Field Crops Res.* 241 107579. 10.1016/j.fcr.2019.107579

[B17] Hu C. L. Sadras V. O. Lu G. Y. Zhang P. X. Han Y. Liu L. (2021). A global meta-analysis of split nitrogen application for improved wheat yield and grain protein content. *Soil Tillage Res.* 213:105111. 10.1016/j.still.2021.105111

[B18] Huang S. H. He P. Jia L. L. Ding W. C. Ullah S. Zhao R. R. (2021). Improving nitrogen use efficiency and reducing environmental cost with long-term nutrient expert management in a summer maize-winter wheat rotation system. *Soil Tillage Res.* 213:105117. 10.1016/j.still.2021.105117

[B19] Jensen L. S. Pedersen I. S. Hansen T. B. Nielsen N. E. (2000). Turnover and fate of N-15-labelled cattle slurry ammonium-N applied in the autumn to winter wheat. *Eur. J. Agron.* 12 23–35. 10.1016/S1161-0301(99)00040-4

[B20] Jiang Z. W. Yang S. H. Chen X. Pang Q. Q. Xu Y. Qi S. T. (2021). Controlled release urea improves rice production and reduces environmental pollution: A research based on meta-analysis and machine learning. *Environ. Sci. Pollut. Res.* 29 3587–3599. 10.1007/s11356-021-15956-2 34392484

[B21] Kamiji Y. Pang J. Y. Milroy S. P. Palta J. A. (2014). Shoot biomass in wheat is the driver for nitrogen uptake under low nitrogen supply, but not under high nitrogen supply. *Field Crops Res.* 165 92–98. 10.1016/j.fcr.2014.04.009

[B22] Khan A. Ahmad A. Ali W. Hussain S. Ajayo B. S. Raza M. A. (2020). Optimization of plant density and nitrogen regimes to mitigate lodging risk in wheat. *Agron. J.* 112 2535–2551. 10.1002/agj2.20211

[B23] Lawrencia D. Wong S. K. Low D. Y. S. Goh B. H. Goh J. K. Ruktanonchai U. R. (2021). Controlled release fertilizers: A review on coating materials and mechanism of release. *Plants(Basel)* 10 238. 10.3390/plants10020238 33530608 PMC7912041

[B24] Liu Y. Liao Y. Liu W. (2020a). High nitrogen application rate and planting density reduce wheat grain yield by reducing filling rate of inferior grain in middle spikelets. *Crop J.* 9 412–426. 10.1016/j.cj.2020.06.013

[B25] Liu Z. Sun K. Liu W. T. Gao T. P. Li G. Han H. F. (2020b). Responses of soil carbon, nitrogen, and wheat and maize productivity to 10 years of decreased nitrogen fertilizer under contrasting tillage systems. *Soil Tillage Res.* 196:104444. 10.1016/j.still.2019.104444

[B26] Lu D. J. Lu F. F. Pan J. X. Cui Z. L. Zou C. Q. Chen X. P. (2015). The effects of cultivar and nitrogen management on wheat yield and nitrogen use efficiency in the North China Plain. *Field Crops Res.* 171 157–164. 10.1016/j.scitotenv.2018.11.126 30447587

[B27] Lv X. K. Ding Y. P. Long M. Liang W. X. Gu X. Y. Liu Y. (2021). Effect of foliar application of various nitrogen forms on starch accumulation and grain filling of wheat (*Triticum aestivum* L.) under drought stress. *Front. Plant Sci.* 12:645379. 10.3389/fpls.2021.645379 33841473 PMC8030621

[B28] Ma G. Liu W. X. Li S. S. Zhang P. P. Wang C. Y. Lu H. F. (2019). Determining the optimal n input to improve grain yield and quality in winter wheat with reduced apparent n loss in the North China Plain. *Front. Plant Sci.* 10:181. 10.3389/fpls.2019.00181 30853966 PMC6396033

[B29] Ma S. C. Wang T. C. Guan X. K. Zhang X. (2018). Effect of sowing time and seeding rate on yield components and water use efficiency of winter wheat by regulating the growth redundancy and physiological traits of root and shoot. *Field Crops Res.* 221 166–174. 10.1016/j.fcr.2018.02.028

[B30] Mackown C. T. Van Sanford D. A. Ma Y. Z. (1989). Main stem sink manipulation in wheat: Effects on nitrogen allocation to tillers. *Plant Physiol.* 89 597–601. 10.1104/pp.89.2.597 16666588 PMC1055887

[B31] Pandey R. K. Maranville J. W. Chetima M. M. (2001). Tropical wheat response to irrigation and nitrogen in a Sahelian environment. II. Biomass accumulation, nitrogen uptake and water extraction. *Eur. J. Agron.* 15 107–118. 10.1016/S1161-0301(01)00097-1

[B32] R Core Team (2021). *R: A language and environment for statistical computing.* Vienna, Austria: R Foundation for Statistical Computing.

[B33] Richards F. J. (1959). A flexible growth function for empirical use. *J. Exp. Bot.* 10 290–301. 10.1093/jxb/10.2.290 12432039

[B34] Salvagiotti F. Miralles D. J. (2008). Radiation interception, biomass production and grain yield as affected by the interaction of nitrogen and sulfur fertilization in wheat. *Eur. J. Agron.* 28 282–290. 10.1016/j.eja.2007.08.002

[B35] Sarker U. Hossain M. N. Iqbal M. A. Oba S. (2020). Bioactive components and radical scavenging activity in selected advance lines of salt-tolerant vegetable amaranth. *Front. Nutr.* 7:587257. 10.3389/fnut.2020.587257 33330589 PMC7734134

[B36] Shadchina T. M. Dmitrieva V. V. (1995). Leaf chlorophyll content as a possible diagnostic mean for the evaluation of plant nitrogen uptake from the soil. *J. Plant Nutr.* 18 1427–1437. 10.1080/01904169509364992

[B37] Shah F. Coulter J. A. Ye C. Wu W. (2020). Yield penalty due to delayed sowing of winter wheat and the mitigatory role of increased seeding rate. *Eur. J. Agron.* 119:126120. 10.1016/j.eja.2020.126120

[B38] Shiferaw B. Smale M. Braun H. J. Duveiller E. Reynolds M. Muricho G. (2013). Crops that feed the world 10. Past successes and future challenges to the role played by wheat in global food security. *Food Secur.* 5 291–317. 10.1007/s12571-013-0263-y

[B39] Si Z. Y. Zain M. Mehmood F. Wang G. S. Gao Y. Duan A. W. (2020). Effects of nitrogen application rate and irrigation regime on growth, yield, and water-nitrogen use efficiency of drip-irrigated winter wheat in the North China Plain. *Agri. Water Manag.* 231:106002. 10.1016/j.agwat.2020.106002

[B40] Sticksel E. Maidl F. X. Retzer F. Fischbeck G. (1999). Nitrogen uptake and utilization in winter wheat under different fertilization regimes, with particular reference to main stems and tillers. *J. Agron. Crop Sci.* 183 47–52. 10.1046/j.1439-037x.1999.00320.x

[B41] Vega-Chacon J. Arbelaez M. I. A. Jorge J. H. Marques R. F. C. Jafelicci M. (2017). pH-responsive poly(aspartic acid) hydrogel-coated magnetite nanoparticles for biomedical applications. *Mater. Sci. Eng. C. Mater. Biol Appl.* 77 366–373. 10.1016/j.msec.2017.03.244 28532042

[B42] Wang D. Xu Z. Z. Zhao J. Y. Wang Y. F. Yu Z. W. (2011). Excessive nitrogen application decreases grain yield and increases nitrogen loss in a wheat-soil system. *Acta Agr. Scand.* 61 681–692. 10.1080/09064710.2010.534108

[B43] Wang H. Y. Zhang Y. T. Chen A. Q. Liu H. B. Zhai L. M. Lei B. K. (2017a). An optimal regional nitrogen application threshold for wheat in the North China Plain considering yield and environmental effects. *Field Crops Res.* 207 52–61. 10.1016/j.fcr.2017.03.002

[B44] Wang X. L. Ye T. Y. Ata-Ul-Karim S. T. Zhu Y. Liu L. L. Cao W. X. (2017b). Development of a critical nitrogen dilution curve based on leaf area duration in wheat. *Front. Plant Sci.* 8:1517. 10.3389/fpls.2017.01517 28928757 PMC5591374

[B45] Wang Q. Y. Tang H. H. Li G. Y. Dong H. Dong X. R. Xu Y. L. (2018). Polyaspartic acid improves maize (*Zea mays* L.) seedling nitrogen assimilation mainly by enhancing nitrate reductase activity. *Agronomy* 8:188. 10.3390/agronomy8090188

[B46] Xu H. C. Dai X. L. Chu J. P. Wang Y. C. Yin L. J. Ma X. (2018). Integrated management strategy for improving the grain yield and nitrogen-use efficiency of winter wheat. *J. Integr. Agri.* 17 315–327. 10.1016/S2095-3119(17)61805-7

[B47] Xu Z. Wan C. Xu X. Feng X. Xu H. (2013). Effect of poly (gamma-glutamic acid) on wheat productivity, nitrogen use efficiency and soil microbes. *J. Soil Sci. Plant Nutr.* 13 744–755. 10.4067/S0718-95162013005000059 27315006

[B48] Yue S. C. Meng Q. F. Zhao R. F. Ye Y. L. Zhang F. S. Cui Z. L. (2012). Change in nitrogen requirement with increasing grain yield for winter wheat. *Agron. J.* 104 1687–1693. 10.2134/agronj2012.0232

[B49] Zadoks J. C. Chang T. T. Konzak C. F. (1974). A decimal code for the growth stages of cereals. *Weed Res.* 14 415–421. 10.1111/j.1365-3180.1974.tb01084.x

[B50] Zhang L. He X. M. Liang Z. Y. Zhang W. Zou C. Q. Chen X. P. (2020). Tiller development affected by nitrogen fertilization in a high-yielding wheat production system. *Crop Sci.* 60 1034–1047. 10.1002/csc2.20140

[B51] Zheng W. K. Liu Z. G. Zhang M. Shi Y. F. Zhu Q. Sun Y. B. (2017). Improving crop yields, nitrogen use efficiencies, and profits by using mixtures of coated controlled-released and uncoated urea in a wheat-maize system. *Field Crops Res.* 205 106–115. 10.1016/j.fcr.2017.02.009

